# Supplementary material for: Development of the ADFICE_IT clinical decision support system to assist deprescribing of fall-risk increasing drugs: A user-centered design approach
Source: PLoS One. 2024 Sep 5;19(9):e0297703. doi: 10.1371/journal.pone.0297703 (PMC11376580; doi:10.1371/journal.pone.0297703)
Supplement: Attachment — Submitted filename: Response to Reviewers.docx [file pone.0297703.s001.docx]

**Response to Reviewers**

We express our gratitude to the editor and reviewers for their insightful feedback, which has been invaluable in enhancing the quality of our manuscript. Below you will find our responses (in **bold**) to each reviewer’s comment together with references to relevant changes we made to the manuscript. The full edited manuscript with tracked changes is attached as well.

**Editor**

**Comment 1**: *Please ensure that your manuscript meets PLOS ONE's style requirements, including those for file naming. The PLOS ONE style templates can be found at* 
[*https://journals.plos.org/plosone/s/file?id=wjVg/PLOSOne_formatting_sample_main_body.pdf*](https://journals.plos.org/plosone/s/file?id=wjVg/PLOSOne_formatting_sample_main_body.pdf) *and* 
[*https://journals.plos.org/plosone/s/file?id=ba62/PLOSOne_formatting_sample_title_authors_affiliations.pdf*](https://journals.plos.org/plosone/s/file?id=ba62/PLOSOne_formatting_sample_title_authors_affiliations.pdf)*.*

**We have revised the manuscript to ensure it meets PLOS ONE’s style requirements.**

**Comment 2**: *We note that the grant information you provided in the ‘Funding Information’ and ‘Financial Disclosure’ sections do not match. When you resubmit, please ensure that you provide the correct grant numbers for the awards you received for your study in the ‘Funding Information’ section.*

**We apologize for this and have updated the ‘Funding Information’ section to match the ‘Financial Disclosure’ section**

**Comment 3**: *Thank you for stating the following financial disclosure:*

*[The ADFICE_IT project is supported by funding from the Netherlands Organization for Health Research and Development (ZonMw, Grant 848017004), The Hague and the Amsterdams Universiteitsfonds: Gepersonaliseerde Medicatieaanpassing bij Oudere Vallers. KMS was supported by US NIH grant T32-GM007592.*

*ZonMw: https://www.zonmw.nl/en*

*Amsterdams Universiteitsfonds: https://www.auf.nl/en*

*NIH: https://www.nih.gov/grants-funding ].*

*Please state what role the funders took in the study. If the funders had no role, please state:* *"The funders had no role in study design, data collection and analysis, decision to publish, or preparation of the manuscript."*

*If this statement is not correct you must amend it as needed.*

*Please include this amended Role of Funder statement in your cover letter; we will change the online submission form on your behalf.*

**Thank you for pointing this out. We have included that the funders had no role in study design, data collection and analysis, decision to publish, or preparation of the manuscript in our cover letter.**

**Comment 4**: *We noted in your submission details that a portion of your manuscript may have been presented or published elsewhere. [This manuscript summarizes the (I) development and (II) feasibility phases of the ADFICE_IT CDSS which consisted of a scoping literature review, systematic literature review, European survey, and semi-structured interviews with physicians, including the aggregation and testing of deprescribing guidelines and the development of the fall-risk prediction model. In phase II, the feasibility of the ADFICE_IT CDSS was tested by means of two usability testing rounds. In this manuscript, we do not report the results from the systematic literature review, European online survey, modified Delphi study, and development of the fall-risk prediction model as these have been published elsewhere by the research team. Instead, we outline how the aggregated results from these studies and others helped inform the final design/development requirements of the CDSS. Thus, we believe that this does not constitute dual publication.] Please clarify whether this publication was peer-reviewed and formally published. If this work was previously peer-reviewed and published, in the cover letter please provide the reason that this work does not constitute dual publication and should be included in the current manuscript.*

**This manuscript has not been previously peer-reviewed or formally published elsewhere. We have expanded on this in our cover letter.**

**Reviewer 1**

**Comment 1:** *The manuscript describes the development of the ADFICE_IT clinical decision support system (CDSS) aimed at assisting physicians in deprescribing fall-risk increasing drugs (FRIDs) to reduce fall risk in older adults. The development process utilized a user-centered design approach involving systematic literature review, surveys, interviews, and usability testing with end users. Overall, the manuscript is a valuable contribution to the field of geriatric care and clinical decision support systems. It provides a robust framework for the development and implementation of similar tools in clinical practice. I only have a few minor questions and advices.*

*The introduction provides a comprehensive overview of the importance of addressing fall risks in older adults and the potential of CDSS to support deprescribing practices. The background is well-researched, citing relevant studies that highlight the complexities of deprescribing FRIDs and the need for decision support tools. The rationale for the study is clearly articulated. The background information is well-supported by recent literature. The objectives of the study are clearly stated, focusing on the development of a CDSS to support the deprescribing of FRIDs and its user-centered design process. The methods section is detailed, describing a multi-phase approach that includes literature reviews, surveys, interviews, and usability testing according to the Medical Research Council framework. Comprehensive description of each phase of the development process is provided.*

**We thank the reviewer for reviewing our manuscript, and for their positive feedback.**

**Comment 2**: *Please explain what you mean with "Dutch fall clinics" as these are not common in all European countries.*

**We thank the reviewer for bringing this to our attention. We have removed the term Dutch fall clinics to avoid confusion and explained the clinical care settings of participating physicians/clinicians:**

*“The latter barriers and facilitators to use analysis was expanded on in the survey (n = 581),* ***which was distributed among European physicians, nurse practitioners, and physician assistants who in their clinical practice (primary, secondary, and tertiary care) see older adults at risk of falling [34].****” p. 10, line 185-188*

*“Clinicians (n = 19) were eligible to participate if they* ***(1)*** ***are working at a hospital in the Netherlands where the ADFICE_IT intervention was set to be implemented and (2) regularly perform a multifactorial fall risk assessment in older adults at risk of falling in an (geriatric) outpatient setting.****” p. 10, line 192-196*

**Comment 3**: *What was the profession of the physicians (geriatrics?)*

**We have addressed this in our response to comment 2, and clarified this in-text in the results section:**

***“A total of 19 clinicians employed by different hospitals in the Netherlands participated of which 15 were geriatric physicians followed by two nurse practitioners, a general practitioner in training and a physical therapist.”*** *p. 16, line 320-322*

**Comment 4**: *The sample size for the usability testing (n = 11) is relatively small, which might limit the generalizability of the findings.*

**We thank the reviewer for this question. Our sample size for the usability testing rounds is in line with sample size recommendations for usability studies. According to research, five participants is enough to uncover 80% of usability problems and each additional participant will uncover fewer and fewer new usability problems, displaying diminishing returns (Alroobae & Mayhew, 2014; Lewis, 1994). Additionally, data saturation was reached after the inclusion of the fifth and sixth physician in the first and second usability testing round, respectively. We have clarified this in-text as follows:**

***“Usability research argues that five participants are sufficient for detecting 80% of a product’s usability problems [49-50].”*** *p. 14, line 273-274*

***“In the first round of usability testing, data saturation was reached after five physicians.”*** *p. 19, line 380-381*

*“After these usability problems were solved, the CDSS underwent the second round of usability testing with physicians (n = 6, three of whom had not participated in the first round).* ***Data saturation was reached after the inclusion of the sixth physician.”*** *p. 19-20, line 386-389*

**References:**

**Alroobaea, R, Mayhew, PJ. How many participants are really enough for**

**usability studies?. 2014 science and information conference. IEEE, 2014.** [**https://doi.org/10.1109/SAI.2014.6918171**](https://doi.org/10.1109/SAI.2014.6918171)

**Lewis, JR. Sample sizes for usability studies: Additional considerations. Human**

**factors. 1994;36(2): 368-378.** [**https://doi.org/10.1177/001872089403600215**](https://doi.org/10.1177/001872089403600215)

**Comment 5**: *More* *detail on the selection criteria for survey (published) and interview (not published) participants would enhance reproducibility.*

**We have addressed this in comment 2 which includes information about the selection criteria for the survey (published) and interview (not published).**

**Comment 6**: *"harmonized dataset (n = 5722) of two Dutch cohorts and a German cohort study of community dwelling older adults (65+)" Please briefly explain and characterize both cohorts.*

**Thank you for pointing out that information about the cohorts is missing. We have provided information about the cohorts in the text as follows on p. 12-13, line 243-252.**

*“The development of the model is described in detail in Van de Loo, Seppala et al. [36], but briefly the model was developed based on a harmonized dataset comprised of two Dutch cohorts and a German cohort study of community-dwelling older adults (65+),* ***namely Longitudinal Aging Study Amsterdam (LASA), B-vitamins for Prevention of Osteoporotic Fractures study (B-PROOF), and Activity and Function in the Elderly in Ulm Study (ActiFE Ulm). For background, LASA is a prospective cohort study to determine predictors and consequences of aging in older adults in the Netherlands [44]. B-PROOF is a Dutch randomized, double-blind, placebo-controlled trial among older adults with an elevated homocysteine concentration [45]. ActiFE Ulm is a population-based cohort study among community dwelling older adults in Ulm and nearby regions in southwestern Germany [46]. From these cohorts, 5722 older adults (65+) for whom medication and follow-up data were recorded were included in the development of the prediction model.”***

**Comment 7**: *The results are presented in a clear and structured manner, detailing the findings from literature reviews, surveys, interviews, and usability testing rounds.*

**We thank the reviewer for their compliments on the results.**

**Comment 8**: *final prediction model consisted of the following 14 predictors: Briefly explain how these variables were measured (e.g. fear of falling, gait speed etc).*

**We have added this information to the results section on p. 17, line 344-351:**

*“The final prediction model consisted of the following 14 predictors: educational status* ***(low, middle, high)****, depression* ***(different validated scales were combined using z-scores)****, body mass index, grip strength* ***(in kg)****, gait speed* ***(in meter per second)****, number of functional limitations* ***(from 0 to 5)****, systolic blood pressure* ***(in mmHg)****, at least one fall in the previous 12 months, at least two falls in the previous 12 months, fear of falling* ***(from 0 = not afraid to 2 = very afraid)****, smoking status* ***(0 = never smoked to 2 = current smoker)****, use of calcium channel blockers, use of antiepileptics, and use of drugs for urinary frequency and incontinence* ***(see Van de Loo, Seppala et al. for harmonization guide) [36].”***

**Comment 9**: *Why did you enter both, one fall and two falls within last 12 months as independent variable in the model?*

**Indeed, both one fall and two falls were included as candidate predictors and both these two predictors were retained in the final prediction model by our variable selection method. We chose to include both variables as candidate predictors because they are both known to have a strong association with the risk of prospective falls.**

**Comment 10**: *prediction model: drugs for urinary frequency and incontinence: what exactly? Only drugs with central side effects or all drugs for urge incontinence?*

**Thank you for this question. The drugs for urinary frequency and incontinence included all the drugs with an ATC code that starts with G04BD plus the individual drug G04CA53. We have instructed readers to view the harmonization guide in Van de Loo, Seppala et al. for more information about the variables used in the prediction model, including the medications as addressed in comment 8.**

**Reference:**

**Van de Loo B, Seppala LJ, van der Velde N, Medlock S, Denkinger M, de Groot	LCPGM, et al. Development of the ADFICE_IT models for predicting falls**

**and recurrent falls in community-dwelling older adults: pooled analyses of**

**European cohorts with special attention to medication. J Gerontol A Biol**

**Sci Med Sci. 2022;77(7):1446-54.** [**https://doi.org/10.1093/gerona/glac080**](https://doi.org/10.1093/gerona/glac080)

**Comment 11**: *The presentation of results from the prediction model development could benefit from more detailed statistical analysis and validation metrics.*

**We agree that the presentation of results from the prediction model is very brief. We have extended the results from the prediction model in-text and refer readers to the literature in which the development and external validation of the prediction model is described in detail:**

*“****Logistic regression with backward elimination was used to develop the model.*** *…* ***Performance was assessed using the C-statistic, whereby a C-statistic value of 0.5 indicates no discrimination and a value of 1 indicates perfect discrimination. Calibration plots were used to assess the agreement between predicted risks and observed outcomes. The prediction model was externally validated in data from geriatric outpatients (refer to Van de Loo, Heymans et al. for detailed methods) [48].****”* p. 13, line 260-267

*“****The results of the prediction model are described in detail in Van de Loo, Seppala et al. [36] and Van de Loo, Heymans et al. [48].*** *…****Performance of the prediction model was comparable to other fall-risk prediction models with a mean C-statistic value of 0.65 in the cohorts used for model development (range 0.61-0.67) [36, 59]. Calibration plots revealed good agreement between the predicted risks and observed outcomes*** ***[35].*** ***External validation of the model showed similar performance in geriatric outpatients as in the cohorts that were used for development, with a C-statistic of 0.66 [48].****”* p. 17, line 343-356

**Comment 12**: *The discussion is well-rounded, addressing the implications of the findings for clinical practice. It acknowledges the limitations of the study and suggests directions for future research.*

**We thank the reviewer for their praise regarding the discussion.**

**Comment 13**: *While limitations are acknowledged, there could be more emphasis on the potential biases introduced by the involvement of the same clinicians in both development and testing phases.*

**Thank you for pointing out that this is unclear for the reader. We have addressed this (in response to another reviewer’s comment) by adding this sentence in the discussion section on p. 24, line 486-489:**

***“Future versions of the CDSS should consider incorporating additional clinicians in the validation process to enhance robustness and, consequently, strengthen the acceptability and applicability of the CDSS recommendations among clinicians.”***

**Again, we much appreciate your thoughtful review of our manuscript. We feel that the manuscript is much improved by these helpful suggestions.**

**Reviewer 2**

**Comment 1**: *Thank you for the opportunity to review this manuscript. This is an important contribution to the literature, giving an overview of the complete development and evaluation processes of a CDSS to assist deprescribing of fall-risk increasing drugs. The results of the multicenter, cluster randomized controlled trial with process evaluation at hospitals in the Netherlands will be of great interest.*

**We thank the reviewer for their positive feedback regarding the development and evaluation processes of the AD*F*ICE_IT CDSS.**

**Comment 2**: *My primary critique of this lengthy manuscript is that it provides primarily previously published data with very limited new information such as the results of the scoping review, presented in 11 lines (209-300) on page 15.*

**We agree with the reviewer that information about the non-published studies should be placed at the forefront of this paper. The primary aim of this manuscript is to summarize the (I) development and (II) feasibility phases of the AD*F*ICE_IT CDSS which consisted of a literature review on risk communication needs, a systematic literature review on barriers and facilitators, European survey, semi-structured interviews with physicians, including two usability testing rounds, the aggregation and testing of the deprescribing guidelines and the development of the fall-risk prediction model. We have no clarified the aim of our manuscript on p. 6, line 140-150 where we outline how the aggregated results from these studies helped inform the final design of the CDSS.**

*“The current paper aims to outline how user and expert insights were used to inform the development of a* ***data-driven*** *CDSS* ***that aims to provide optimal support for physicians during the deprescribing of FRIDs by generating a personalized fall-risk estimate and guideline-based medication advice tailored to the health conditions of each individual patient. Following a user-centered design approach****, we showcase how users were successfully involved in different stages of the development process to increase the system’s acceptance and adoption in its intended clinical care setting later on.* ***This CDSS is part of the ADFICE_IT (Alerting on adverse Drug reactions: Falls prevention Improvement through developing a Computerized clinical support system: Effectiveness of Individualized medicaTion withdrawal) intervention, a novel deprescribing intervention that aims to prevent medication-related falls in older adults by means of a CDSS for clinicians and an online portal for patients [31].”***

**Moreover, in response to your comments and those from other reviewers, we have expanded on the literature review on risk communication needs (comments 6 and 12), semi-structured interviews (comments 7 and 13), and usability testing rounds (comments 11, 18 and 20). We have added additional information about the development of the prediction model based on comments from another reviewer. However, the results from the systematic review on barriers and facilitators, European survey, and modified Delphi study are not reported in this manuscript.**

**Comment 3**: *Despite the appreciated summary in table 1, the many components described in the article required multiple back and forth between the methods and results sections.*

**We thank the reviewer for pointing this out. We have now ensured that the methods and results sections for relevant studies are aligned, and have adapted this in the manuscript as illustrated in our response to comments 6, 7, 12, 13, 18 and 19.**

**Comment 4**: *A few specific questions / comments arose as I reviewed the manuscript: P.4. line 71, the study registration number could be provided.*

**We thank the reviewer for this suggestion. The trial is registered with ClinicalTrials.gov. We have included this information in the abstract:**

***“The trial is registered with ClinicalTrials.gov (date; 7-7-2022, identifier: NCT05449470).”***

**Comment 5**: *P.7 line 157, the patient portal is only mentioned here and briefly in the discussion. I suggest to either delete or develop further.*

**We agree that the patient portal is mentioned only briefly. However, we feel it is important to retain the brief mention of it in this paper, to place the CDSS described in this paper in the context of the larger AD*F*ICE_IT intervention, which includes the patient portal. We have clarified this by revising the aim of this manuscript as shown in our response to comment 2.**

**Comment 6**: *P.10, lines 190-191, the sentence ‘The scoping literature review assessed the risk communication needs of physicians.’ is the only methods information I found for this component. This is insufficient to describe the methods of a scoping review.*

**We thank the reviewer for this remark. We have included information about the methods of our literature review on p. 10, line 178-184. Upon consideration, we have decided not to classify our work as a scoping review, as the methods we used for selecting appropriate literature related to the needs assessment aligns more closely with a standard literature review:**

*“The literature review assessed the risk communication needs of physicians.* ***Literature for the review was retrieved from Google Scholar using the following search terms: “doctor,” “physician,” “healthcare provider,” “clinical decision support system,” “clinical support system,” “satisfaction,” “preferences,” “usability,” “user centered design,” “risk information,” “risk communication,” “medication,” “drug,” “drug on drug.” References were excluded if (1) the sample did not compromise of physicians/doctors; (2) the study did not discuss a medication-related CDSS; (3) the study was not published between 2014 and 2020.”***

**Comment 7**: *P.11, line 194, more information on the selection process of the participating physicians (GP, specialists, how they were selected…) is essential.*

**We agree the selection process of the participating health care providers is limited. We have clarified this in the methods on p. 10, line 185-196:**

*“The latter barriers and facilitators to use analysis was expanded on in the survey* ***(n = 581), which was distributed among European physicians, nurse practitioners, and physician assistants who in their clinical practice (primary, secondary, and tertiary care) see older adults at risk of falling*** *[34].* ***In-person****, semi-structured interviews with* ***clinicians were conducted to*** *assess the need for effective implementation and trustworthy decision making* ***with the help of a topic-list developed for this study.*** *…****Clinicians (n = 19) were eligible to participate if they (1) are working at a hospital in the Netherlands where the ADFICE_IT intervention was set to be implemented and (2) regularly perform a multifactorial fall risk assessment in older adults at risk of falling in an (geriatric) outpatient setting. All interviews were conducted in Dutch, voice-recorded, and transcribed verbatim.”***

**Comment 8**: *P.13, line 238, 10 patients seem minimal. Enough to cover the wide scope of recommendations?*

**Thank you for pointing this out. We agree that 10 patients seems like a small number, but these are rather complex patients and it does not take many patients to get a fairly long list of medications. We have added the following text to the results section on p. 19, line 374 to explain this:**

***"The 10 patients were taking 42 different medications, triggering 50 distinct rules."***

**About 1/3 of the rules cover rare medications/conditions and thus are not expected to fire often, so this covers about half of the rules for common medications/conditions. All of the rules were covered in the previous step (using fictional patients, specifically formulated to trigger all of the rules).**

**Comment 9**: *P.13, line 259, will the system be used by other physicians such as GP?*

**We thank the reviewer for this question. The primary end users of the system are geriatric physicians. We have clarified this in the text as follows on p. 14, line 271-273:**

*“This was achieved through two usability testing rounds with geriatric physicians (n = 11) from Dutch hospital****s*** ***(i.e., the primary users of the system).”***

**Comment 10**: *Could usability differ between GP and geriatric physicians?*

**Thank you for this question. Our CDSS has been particularly developed for use in the hospital setting, primarily among geriatric physicians treating older fall risk patients. Therefore, we did not study differences in usability between GPs and geriatric physicians.**

**Comment 11**: *P.14, lines 260-261, they were presented 3 cases but only completed at least one?*

**This is indeed unclear, thank you for pointing this out. Each participant was presented with three cases and used the same CDSS task scenarios (i.e., scripted navigation) to complete each of the cases. This has been corrected in the methods on p. 14, line 274-278:**

*“Each physician was presented with three hypothetical patient cases that varied in fall risk (e.g., high versus low risk). Next, physicians were asked to carry out a scripted navigation* ***for each case, which*** *consisted of realistic CDSS task scenarios, resembling aspects of clinical documentation (e.g., analyzing a patient’s fall-risk, selecting relevant treatment options, making a referral).”*

**Comment 12**: *P.15, lines 289-300, I do not have enough methods information to appreciate the results of the scoping review.*

**We thank the reviewer for this comment. We have elaborated on this in our response to comment 6. Additionally, we have extended it with the following text in the results on p. 16, line 316-317:**

***“The aim of the literature review was to identify the risk communication needs of physicians. A total of six articles were included.”***

**Comment 13**: *P.15, lines 301-312, I do not have enough information. For example, the reader must go back to the abstract for the n=19. Who were those physicians? How were the interviews conducted? Was a standard questionnaire used?*

**We have elaborated on the semi-structured interviews in our response to comment 7. The interviews were conducted in-person in Dutch, voice-recorded and transcribed verbatim. A topic-list was developed to help guide the interviews. Participating clinicians were employed by hospitals in the Netherlands who regularly conducted a multifactorial fall risk assessment in older adults at risk of falling in an (geriatric) outpatient setting. Additionally, we have now clarified this in the results section on p. 17, line 318-322:**

***“The aim of the interviews with clinicians was to identify the information-related needs for effective and trustworthy decision making, and the requirements for successful implementation of the CDSS in practice. A total of 19 clinicians employed by different hospitals in the Netherlands participated of which 15 were geriatric physicians followed by two nurse practitioners, a general practitioner in training, and a physical therapist.”***

**Comment 14**: *P.16, line 314, how was this hierarchy decided?*

**We thank the reviewer for this question. We have removed the term hierarchy from the manuscript to avoid confusion as we aim to highlight the systematic approach we used to aggregate the needs of clinicians obtained from both literature reviews, the European online survey, and the semi-structured interviews with physicians. We adjusted this in-text on p. 16-17, line 335-337 and in Table 2.**

*“Table 2 shows the aggregated system needs* ***of clinicians obtained*** *from the literature review* ***on risk communication needs, systematic review on barriers and facilitators,*** *European online survey, and semi-structured interviews with clinicians.”*

**Comment 15**: *P.16, lines 321-325, are all these variables (e.g. educational status, grip strength, fear of falling) readily available in a majority of EHR?*

**Thank you for this question. Variables such as educational status, grip strength, and fear of falling are tests/questions that are part of a comprehensive geriatric assessment or multifactorial fall risk assessment and are thus readily available in the majority of EHRs, especially at hospitals with a high frequency of at risk older adult fallers. We have adjusted this in text on p. 14, line 256-260.**

*“Candidate predictors were selected based on previously reported risk factors for falls that are easily obtained* ***from the Electronic Health Record (EHR)****, such as sociodemographic variables; measures of emotional, cognitive and physical functioning; self-reported chronic conditions; variables related to lifestyle; biomarkers; and use of certain medications.”*

**Comment 16**: *P.18, line 336, was ‘input from external expert developers’ received?*

**Yes. Two of the three primary developers contributed specifically because this is an open source project, and we also received help and advice from several other developers. We have added "see acknowledgements" in the text on p. 19, line 366-367 to draw attention to the contributions of these developers.**

*“Development as open source software facilitated seeking and incorporating input from external expert developers* ***(see acknowledgements).”***

**Comment 17**: *P.18, lines 343-345, what are the numbers? 95% and 100% agreement on what?*

**We agree that this was unclear. We have adjusted the text in the results section on p. 19, line 374-375 as follows:**

*"This resulted in 100% agreement* ***with these recommendations*** *from one clinician and 95% agreement from the other, which exceeded the minimum threshold of 90% agreement."*

**Comment 18**: *p. 18, line 348, ’74 individual usability problems’. What is the context? The three hypothetical cases? How many of those cases were evaluated by the 5 physicians?*

**Thank you for pointing out that terminology used for reporting the results of the usability studies is unclear. We have made the following changes in-text on p. 19, line 381-383 including those from our response to comment 11.**

*“The physicians (n = 5) identified* ***a total of*** *74 usability problems* ***with the CDSS, with a*** *mean Nielsen’s severity rating classification* ***of 2.49*** *(i.e., between minor to major usability problems) [52].”*

**Comment 19**: *P. 18, line 354, I have to go back to the abstract to find the n=11 and understand that these are 6 new physicians.*

**Thank you for pointing this out. We have clarified this in the results as follows on p. 19, line 379-389:**

*“****To identify usability problems early on, the prototype of the CDSS was evaluated in two separate usability testing rounds with geriatric physicians. In the first round of usability testing, data saturation was reached after five physicians.*** *…* ***After these usability*** ***problems were solved,*** *the CDSS underwent* ***the*** *second* ***round of*** *usability testing* ***with physicians (n = 6, three of whom had not participated in the first round). Data saturation was reached after the inclusion of the sixth physician.”***

**Comment 20**: *P.18, line 355, give the number of problems instead of the 16%.*

**We thank the reviewer for this suggestion. We have adjusted this in text on p. 20, line 396-398:**

*“The number of* ***total*** *usability problems identified by* ***physicians was 11*** *and the mean Nielsen’s severity rating was 1.87 (i.e., between cosmetic to minor problems) [52].”*

**Comment 21**: *P.19, lines 364, the figures were unreadable. Unfortunately, this portion of the manuscript could not be assessed.*

**We would like to apologize for submitting unreadable figures in the first version of our manuscript. We have submitted new figures with this version and enhanced the quality of the figures.**

**We would like to thank you again for your thoughtful review of our manuscript. We believe your helpful remarks have led to a much improved version of the manuscript.**

**Reviewer 3**

**Comment 1**: *The manuscript presents a technically sound piece of scientific research that describes the development and evaluation of the ADFICE_IT clinical decision support system (CDSS) designed to assist in the deprescribing of fall-risk increasing drugs (FRIDs) in older adults. The study is well-grounded, with data supporting the conclusions drawn. The experiments were conducted rigorously, with appropriate controls, replication, and sample sizes.*

*User-Centered Design Approach: The involvement of end users throughout the development process ensures that the CDSS is practical, useful, and likely to be accepted in clinical settings. This approach has been shown to improve technology adoption by healthcare professionals.*

*Integration of Deprescribing Guidelines and Risk Prediction Models: The CDSS leverages a combination of deprescribing guidelines and a validated fall-risk prediction model, which addresses the complexity involved in deprescribing FRIDs. This systematic approach can significantly aid physicians in making informed decisions to reduce fall risks in older adults.*

*Iterative Development and Usability Testing: The use of agile methodologies and iterative feedback from usability testing rounds allowed the authors to refine the CDSS continuously. This resulted in a user-friendly system with minimized usability barriers, enhancing its effectiveness and efficiency in clinical practice.*

*Detailed Validation Process: The CDSS underwent thorough verification and validation testing, including both unit tests and real patient data validation. This rigorous process ensured the reliability and accuracy of the system's recommendations.*

**Thank you for your positive evaluation of the manuscript.**

**Comment 2**: *Scalability: Although clarity of code was prioritized over efficiency and scalability, future versions of the software should consider enhancing scalability to support a higher number of concurrent users. This will be particularly important for broader implementation across multiple clinical settings.*

**We agree that scalability is important for the future. The current version of the software is sufficient for the current use case, but should be improved (for example by reducing the number of database queries needed per patient) if it is extended for use by a broader audience than only Dutch geriatricians. We have added this to the discussion on p. 23, line 478-479:**

***“Future versions of the software should consider enhancing scalability to support a higher number of concurrent users.”***

**Comment 3**: *Broader Validation: While the involvement of one clinician in the early validation steps was beneficial, incorporating a more diverse group of clinicians in the initial stages could further strengthen the robustness of the validation process. This would ensure that the CDSS recommendations are universally acceptable and applicable.*

**We agree that broader validation is important for ensuring the acceptance and applicability of the CDSS in the future. We have added this to the discussion on p. 24, line 484-487:**

***“Future versions of the CDSS should consider incorporating additional clinicians in the validation process to enhance robustness and, consequently, strengthen the acceptability and applicability of the CDSS recommendations among clinicians.”***

**Comment 4**: *Comprehensive Documentation: Providing more detailed documentation and user guides could facilitate easier implementation and training for new users. This would help in overcoming initial barriers to adoption and ensure that users can fully utilize the system's capabilities.*

**We thank the reviewer for this comment. A user guide is incorporated within the CDSS. We have added this information in the manuscript on p. 20, line 400-406:**

*“Page 1 (titled “Start” in fig. 1) is the landing page of the CDSS. On this page the physician can…****; and (4) view a user guide of the system (titled “Handleiding”).”***

**Moreover, a training for physicians has been developed and is currently being tested as part of the AD*F*ICE_IT intervention.**

**Comment 5**: *The manuscript is well-written and presented in clear, standard English. The data underlying the findings are fully available, adhering to PLOS ONE's data availability policy. The development and evaluation of the ADFICE_IT CDSS represent a significant advancement in supporting the safe deprescribing of FRIDs to prevent falls in older adults. The user-centered design approach and rigorous testing processes enhance the system's practicality and reliability.*

*The manuscript's clear and systematic presentation, combined with the robustness of the research methods, makes it a valuable contribution to the field. It addresses a critical aspect of geriatric care, and its findings have the potential to improve patient outcomes significantly.*

*The manuscript presents a significant and well-executed study on the development and evaluation of the ADFICE_IT clinical decision support system (CDSS) for assisting in the deprescribing of fall-risk increasing drugs (FRIDs) in older adults. The study is technically sound, with robust data supporting the conclusions, and it follows rigorous experimental protocols. The user-centered design approach and iterative usability testing have resulted in a practical and user-friendly system that addresses a critical aspect of geriatric care.*

**Thank you for your compliments surrounding the methodologies and evidence base used for developing the AD*F*ICE_IT CDSS.**

**Comment 8**: *However, there are a few areas that could benefit from further improvement before publication. Scalability: The manuscript should address potential scalability issues and discuss plans for future enhancements to support a higher number of concurrent users.*

**We have addressed issues related to scalability in our response to comment 2.**

**Comment 6**: *Comprehensive Documentation: Providing more detailed documentation and user guides will facilitate easier implementation and training, helping to overcome initial adoption barriers.*

**Thank you for pointing this out. We provided information about a user guide and training in our response to comment 4.**

**Comment 7**: *Alignment with Standards: Consider the pertinence of describing in the manuscript how the ADFICE_IT CDSS aligns with the quality characteristics defined in ISO/IEC 25010:2011. Including this information can strengthen the manuscript by demonstrating that the CDSS meets recognized benchmarks for software quality, thus enhancing its credibility and reliability. If the authors can access this standard, it is recommended to review it to ensure comprehensive alignment.*

**Unfortunately, we do not have access to this standard. We do have access to the summary offered on the ISO website and third-party guidance based on this standard (ISO/IED 25010:2023). The steps we describe in this paper align closely with the quality lifecycle model described in these summaries (assessment of functional suitability, performance, compatibility/interoperability, usability, reliability, security, maintainability, and portability). We have added this to the paper. Since, we fully agree that this is important, and have added it to the discussion on p. 24, line 489-491:**

***“Additionally, while the steps described in this paper closely align with the quality lifecycle model described in the ISO/IED 25010:2023 standard, standards such as ISO/IEC 25010 could help guide improvements to the software.”***

**Again, we would like to thank you for your careful review of our manuscript. We believe the manuscript has much improved by your helpful suggestions.**
